# Epidermal Growth Factor Receptor Inhibitors: A Review of Cutaneous Adverse Events and Management

**DOI:** 10.1155/2014/734249

**Published:** 2014-03-02

**Authors:** K. Chanprapaph, V. Vachiramon, P. Rattanakaemakorn

**Affiliations:** Division of Dermatology, Faculty of Medicine Ramathibodi Hospital, Mahidol University, 270 Rama VI Road, Rajthevi, Bangkok 10400, Thailand

## Abstract

Epidermal growth factor inhibitors (EGFRI), the first targeted cancer therapy, are currently an essential treatment for many advance-stage epithelial cancers. These agents have the superior ability to target cancers cells and better safety profile compared to conventional chemotherapies. However, cutaneous adverse events are common due to the interference of epidermal growth factor receptor (EGFR) signaling in the skin. Cutaneous toxicities lead to poor compliance, drug cessation, and psychosocial discomfort. This paper summarizes the current knowledge concerning the presentation and management of skin toxicity from EGFRI. The common dermatologic adverse events are papulopustules and xerosis. Less common findings are paronychia, regulatory abnormalities of hair growth, maculopapular rash, mucositis, and postinflammatory hyperpigmentation. Radiation enhances EGFRI rash due to synergistic toxicity. There is a positive correlation between the occurrence and severity of cutaneous adverse effects and tumor response. To date, prophylactic systemic tetracycline and tetracycline class antibiotics have proven to be the most effective treatment regime.

## 1. Introduction

New chemotherapeutic agents have been developed with increased understanding of the pathogenesis of malignant tumors. Treatments of many epithelial cancers have focused on attacking specific inhibitors of oncologic molecules. These agents have improved ability to target cancers cells and enhance safety profile compared to conventional chemotherapies. Despite the benefits, targeted chemotherapies have enormous skin adverse events, which may lead to poor adherence, dose interruption, and discontinuation of these therapeutic regimens. Moreover, psychosocial discomfort leading to reduction in the quality of life can frequently occur. However, the presence and severity of cutaneous toxicity has shown to have positive correlation with patient survival and could be a surrogate marker for tumor response, especially for the epidermal growth factor receptor inhibitors (EGFRI). Optimum management is essential and will allow enabling patients to remain on these life prolonging therapies.

This paper summarizes the current knowledge concerning the presentation and management of skin toxicity from targeted chemotherapy, giving emphasis on the single-targeted inhibitor, EGFRI. It is based on published article from Medline database. The reports on prevalence and severity of skin side effects are based on prospective and retrospective studies and clinical reviews. The management of targeted chemotherapy which induced skin toxicity can be divided into prophylactic and treatment measures. Prophylactic treatments are reviewed under the consensus of few randomized control trials. However, as far as specific treatment for cutaneous toxicity is concerned, evidence based treatments are lacking and recommendations from weaker sources, for example, uncontrolled trials and expert recommendations, have been utilized.

## 2. Epidermal Growth Factor Receptor Inhibitors

Human epithelial cancer cells are distinguished by the functional activities of growth factors and their receptor, mainly of the epidermal growth factor receptor (EGFR) family. It belongs to a family receptor named tyrosine kinase. Overexpression of EGFR promotes gene amplification and mutation consequence in cell proliferation, survival, invasion, metastasis, and tumor induced neoangiogenesis [1]. EGFR inhibitor was the first agent developed as a target cancer therapy. Two classes of EGFR inhibitors are in current use: the monoclonal antibodies (cetuximab, panitumumab, and matuzumab) that target the extracellular ligand-binding domain and small-molecule tyrosine kinase inhibitors (gefitinib, erlotinib, lapatinib, and afatinib) which target intracellular domain [1, 2]. EGFR inhibitors have been approved for the treatment of metastatic non-small-cell lung cancer, colorectal cancer, pancreatic cancer, and squamous cell carcinoma of the head and neck [1]. When the expression of EGFR is decreased, inhibition of downstream signaling occurs in malignant tumor cells. This results in inhibition of metastasis, growth, proliferation, differentiation, and angiogenesis and causing apoptosis of cancer cells [2].

Unlike conventional chemotherapy that generally targets rapidly dividing cells by interfering with DNA and RNA synthesis, EGFR inhibitors have favorable systemic adverse events. However, EGFR is crucial for the normal development and physiology of the skin. It is highly expressed in the epidermis especially in the basal cell layer, the outer root sheath of hair follicles, and the sebaceous epithelium. It is also moderately expressed in the eccrine epithelium and dendritic antigen-presenting cells. Therefore, clinically distinct patterns of cutaneous toxicity of EGFR inhibitors can be observed from alteration of the normal function of these structures. Cutaneous eruptions are considered as drug class-specific. Wide range dermatologic adverse events can be found. The common findings are papulopustules and xerosis. Less common side effects are paronychia, regulatory abnormalities of hair growth, maculopapular rash, mucositis, and postinflammatory hyperpigmentation.

## 3. Clinical Findings of Dermatologic Adverse Events

The earliest and most common cutaneous adverse events occurring from 50 to 100% of the reported clinical trials are papulopustular rash, sometimes referred to as acneform eruption [3–6]. They usually develop within the first weeks of treatment and can occur as early as 2 days and as late as 6 weeks after EGFR inhibitors have commenced [7].

Typical presentations comprise erythematous follicular centered papules, pustules with absence comedones. Lesions can be painful and pruritic [8].

**Figure 1:**
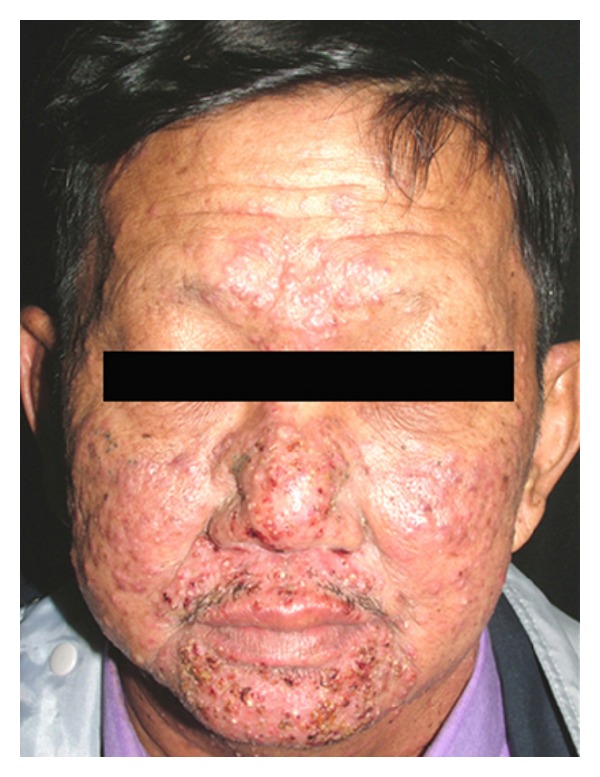
Papulopustular eruption. A 52-year-old man with non-small-cell lung carcinoma stage IV developed papulopustules 6 days after erlotinib was commenced.

Because EGFRs are highly expressed in sebaceous epithelium, eruptions are generally presented in seborrheic areas involving the scalp, face, neck, chest, and upper back (Figure 1). Involvement of the extremities, lower back, abdomen, and buttocks can also occur. Periorbital region and the palms and soles are usually spared [9].

The pathogenesis behind EGFRI induced papulopustules is marked alterations in growth, differentiation of the epidermis leading to altered corneocyte terminal differentiation. Compact orthokeratosis and dyskeratosis of the epidermis can be seen in both the affected and unaffected skin [10]. Other major changes are damages of the sebaceous glands and follicular infundibula which generate cytokine release as well as inflammatory cell infiltration in periappendageal areas. Dermal neutophilic suppurative infiltrations without evidence of infections are seen at the onset of papulopustular rash [7, 10]. The initial reaction is considered as sterile folliculitis, supporting that microorganisms are not the major cause of folliculitis. However, through time, presence of secondary infection may occur from compromised epidermal barrier. Retrospective studies and case series have shown some evidence of dermatologic infection, mainly bacterial, at sites previously affected by dermatologic toxicity for EGFRI [11–13]. This enhances the value of antibiotic treatment, as well as routine bacterial cultures on papulopustular rashes.

Papulopustular eruptions associated with monoclonal antibodies tend to be more severe and widespread compared to small-molecule tyrosine kinase inhibitors [14]. Regardless of the offending agent, lesions will decrease in intensity over several weeks but persist as mild erythema and follicular papules throughout the course of treatment [15, 16].

**Figure 2:**
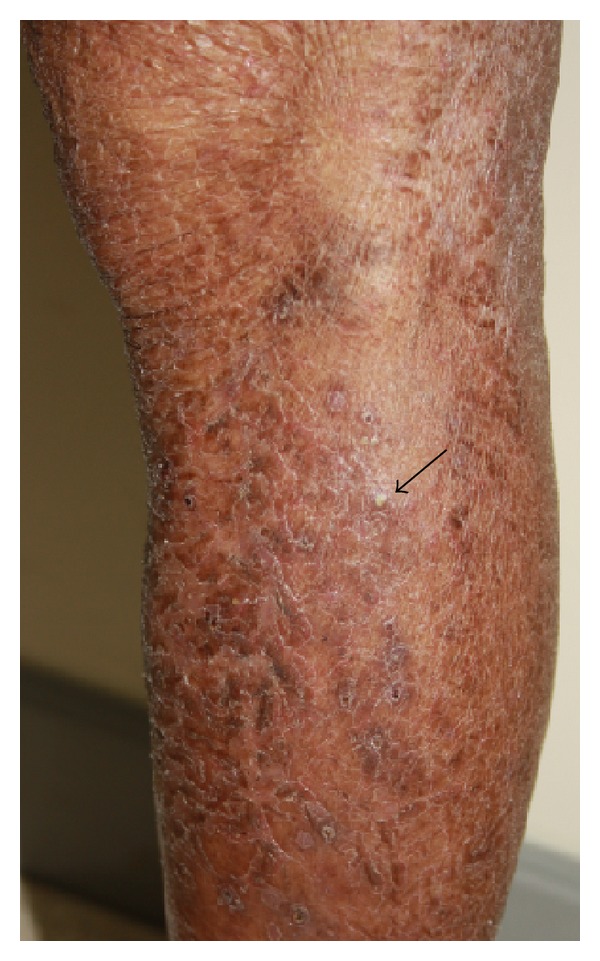
Xerosis. Ill-defined dried scaly patch with mild erythema on the left leg, occurring 3 weeks following gefitinib. Notice scattered pustules, showing evidence that xerosis took place where papulopustules have developed.

Xerosis is the second most common cutaneous adverse event from EFFRI, occurring from over 35% in most reports. It has also shown to be the leading skin adverse event in a few reports, prevalence of approximately 50% to 100% [17, 18]. Older patients with prior exposure to cytotoxic agents leading to alteration in skin barrier are prone to develop dry skin. Xerosis presents as dry, itchy, scaly patches which may progress to painful fissuring and xerotic eczema. It may take place at sites where papulopustules have developed; however, more widespread involvement usually occurs (Figure 2) [7, 8].

Paronychia is a less common side effect described in 5–20% [17–20]. It usually presents as painful periungual inflammation. Paronychia, which involves many fingers and toes, is particularly disturbing when the finger nails are affected [7]. In severe cases, ingrown nail, periungual absess, and pyogenic granuloma-like lesions can occur. Paronychia usually develops later, approximately after 1-2 months. The pathogenesis remains unclear, but it is proposed that EGFRI may directly inhibit keratinocytes in the nail matrix [7]. Infection is not the main culprit of paronychia, as Staphylococcus aureus was cultured in a few patients and they were unresponsive to antistaphylococcal antibiotics [21].

**Figure 3:**
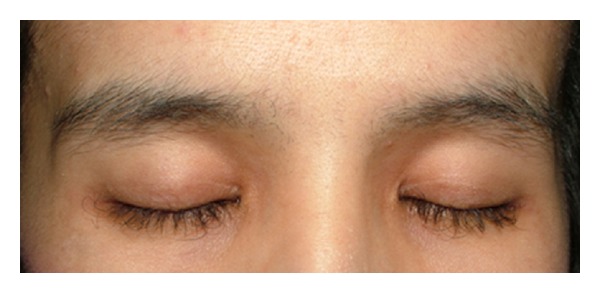
Trichomegaly. Trichomegaly developed in a 40-year-old woman, 3 months preceding erlotinib. Notice the wavy, curly, and aberrant elongation of the eyelashes.

Regulatory abnormalities of the hair growth can infrequently occur. Hair overgrowth such as trichomegaly and hypertrichosis have been described, the former being more relevant clinically. Trichomegaly usually develops 2–5 months after initiating EGFRI. It is relatively rare but can have significant esthetic damage. Eyelashes will appear wavy, curly, and aberrant (Figure 3). This may lead to corneal irritation and ultimately ulceration. The pathogenesis is hypothesized to be from increased terminal differentiation from EGFR inhibition [7].

**Figure 4:**
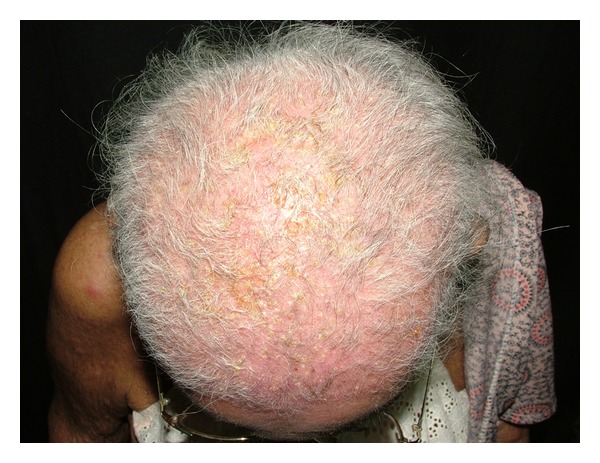
Scalp pustule and scaring alopecia. A 78-year-old woman developed follicular centered pustular eruption on the scalp and scaring alopecia after 3 months of erlotinib.

Follicular pustules may infrequently occur [22]. Extensive scalp pustules may lead to scaring alopecia (Figure 4). Hair loss both scarring and nonscarring inflammatory alopecia have also been reported [23, 24]. The precise mechanism for inflammatory hair loss is unclear but may possibly reflect severe endpoint of the follicular papulopustular eruptions [24]. Hair curling and rigidity and hair repigmentation or depigmentation have been reported [25].

Other less common cutaneous sides are maculopapular rash, mucositis, and postinflammatory hyperpigmentation [7, 17].

## 4. Severity Grading System

There are many proposed criteria to grade the severity of cutaneous toxicity from EGFRI. By far the most commonly used one is the system developed by the US National Cancer Institute in the catalog of common toxicity criteria (NCI-CTC version 4.0) Grade 1: papules and/or pustules covering <10% of the body-surface area (BSA) with or without symptoms of pruritis or tenderness, Grade 2: papules and/or pustules covering 10–30% of the BSA with or without symptoms of pruritis or tenderness; with psychosocial impact, Grade 3: papules and/or pustules covering >30% of the BSA with or without symptoms of pruritis or tenderness; limiting self-care activities of daily living, associated with local superinfection with oral antibiotics indicated, Grade 4: covering any percentage of the BSA with or without symptoms of pruritis or tenderness; associated with extensive super-infection with intravenous antibiotics indicate; life-threatening consequences, and Grade 5: Death [26].

## 5. Skin Toxicity and Tumor Response

Evidence has revealed that tumor response and patient survival have improved in the present and increased severity rash from EGFR inhibitors [27]. Cutaneous toxicity is currently considered as a surrogate marker for tumor response as well as overall survival [28]. Moreover, patients experiencing multiple cutaneous toxicity had better therapeutic outcome compared to single skin adverse event [17]. Frequency and severity of skin rashes are dose dependent [29]. Therefore, gradual dose increment until the skin eruptions appears is a strategy to maximize efficacy of EGFR inhibitors.

## 6. The Effect of EGFRI and Concurrent Ionized Radiation

**Figure 5:**
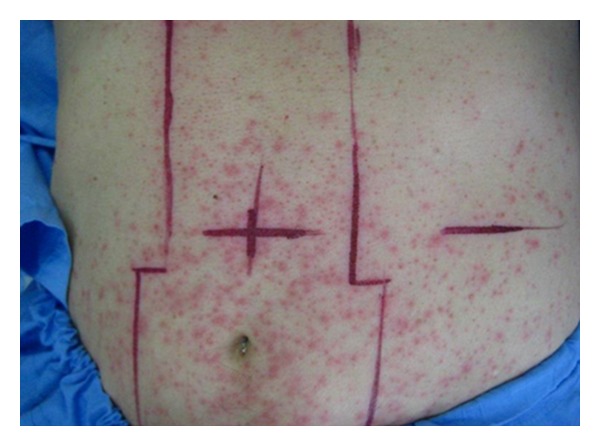
Papulopustules on irradiated area. A 65-year-old-man with non-small-cell lung cancer stage IV and cauda equina syndrome was admitted for radiation. Erlotinib was given 7 days ago. After 2 days of radiotherapy he developed papulopustular eruption predominantly on the irradiation field.

Patient receiving EGFRI have advance stage carcinoma and frequently require radiation in addition to chemotherapy. The effect of concurrent ionized radiation and EGFRI can be categorized into early and late phase. Initially, when EGFRI is commenced in the same period as radiation compared to radiation alone, the ratio for radiation dermatitis as well as EGFRI side effects increases. EGFRI eruption occurs predominantly in the irradiated areas (Figure 5). These agents have synergistic cytotoxicity as well as therapeutic response. Radiation upregulates EGFR in the normal skin; hence, the presence of EGFRI rash accelerates [30–33]. These cutaneous side effects may also lead to treatment interruption. Late actions of EGFR inhibitors and ionized radiation are totally different from the early phases of enhance cutaneous side effects. With prolong irradiation there is absence of skin toxicity to EGFR inhibitors in the preirradiated area. This is due to the fact that radiation induces depletion of basal layer stem cells by apoptosis. Moreover, late chronic radiation causes loss of hair follicles and sebaceous glands by TGF-beta mediated fibrosis [34].

## 7. Management of EGFRI Induced Skin Toxicity

The management of EGFRI which induce skin toxicity can be categorized into prophylaxis and reactive treatment. There are several well-designed randomized control trials (RCT) on agents that could possibly prevent or alleviate symptoms of cutaneous toxicities given prior to EGFRI. However, there are only a few uncontrolled trials, case series, and case reports for reactive treatment of EGFRI-associated dermatologic adverse event.

## 8. Prophylactic Treatment

### 8.1. Antibiotics: Tetracycline and Tetracycline-Class

To date, there are 4 published randomized control and 1 meta-analysis on the use of antibiotics, all comprising tetracycline and the tetracycline-class (Table 1).

The first published clinical trial on the prophylaxis of EGFRI induced papulopustule was done by Scope et al. This was a randomized double-blinded trial on prophylactic oral minocycline and topical tazarotene for papulopustules from cetuximab. The group of patients who received minocycline showed benefit initially, during weeks 1 to 4, with less development of facial lesions and lower itch severity. After the 1st month this advantage was no longer evident [35]. Another randomized control trial by Jatoi et al. compared the efficacy of prophylactic oral tetracycline versus placebo on the incidence and severity of rash from EGFRIs. The presence of rash was the same in the 2 groups. However, the severity was significantly lower in the tetracycline group during the first 4 weeks of treatment [36].

Lacouture et al. published a trial on patients receiving panitumumab-containing therapy. Participants were randomly assigned to receive either prophylactic or reactive treatment. Prophylactic treatment comprised using skin moisturizers, sunscreen, 1% hydrocortisone cream, and doxycycline. The reactive treatment meant any kind of treatment necessary following skin side effects. The results revealed that their prophylactic regimen (doxycycline arm) could decrease the incidence of ≥2 grade skin toxicity compared to the reactive treatment [37].

Deplanque et al. conducted a large randomized clinical trial to access the effect of doxycycline in reducing the incidence and severity of erlotinib-induced folliculitis during 4 months of treatment. The results showed that the incidence and severity of folliculitis were significantly less in the doxycycline arm compared to placebo. Moreover, doxycycline was associated with decrease in severity of other cutaneous adverse events [38].

A meta-analysis on antibiotic as a prophylactic regimen for skin rash concluded that antibiotics did not reduce the incidence of rash from EGFRI. However, the relative risk for severity of rash was reduced by 42% to 47% with the use of antibiotics [39].

## 9. Topical Treatments

Up until now, there have been several control trials on the prophylactic use of topical agents, one for pimecrolimus, one for tazarotene, and one for sunscreen. There has also been one uncontrolled trial for the preventive measures of topical vitamin K1 for EGFRI rashes (Table 2).

The preventive effect of tazarotene was evaluated in parallel to minocycline for patients receiving cetuximab by Scope et al. Tazarotene was allocated randomly to apply on either the left or right side of the face. This study showed that tazarotene caused significant irritation and gave no benefit in preventing the rash. The rash was even assessed as more severe in the tazarotene side in 10% of the patients. Therefore, this agent is not recommended [35].

Scope et al. conducted a haft face study to evaluate whether pimecrolimus could reduce acne-like eruption as well as rash severity induced by cetuximab. After 2 weeks, lesion counts were significantly less in the pimecrolimus treated side. This benefit was maintained to week 5. However, there was a trend towards lesion decrement on both sides. Moreover, no significant difference in rash severity and patient assessment of symptoms was observed. Therefore, pimecrolimus did not achieve significant clinical benefit [40].

Vitamin K, a phosphatase inhibitor, and one of the most potent EGFR activators, was evaluated as another prophylactic agent in patients receiving cetuximab combined with chemotherapy. This was an uncontrolled study on Vitamin K1 analog. Vitamin K1 cream had shown to prevent high grade cutaneous side effect. None of the patients developed Grade 3 or 4 toxicity which should normally develop in 20% of patient receiving cetuximab [41].

The effectiveness of sunscreen in the prevention EGFRI induced rash was conducted by Jatoi et al. Patients receiving various types of EGFRIs were randomly assigned to receive either twice daily sun protecting factor 60 sunscreen or placebo for 4 weeks. There was no significant difference in rash severity or patient-reported outcome in both groups. Moreover, application of sunscreen did not cause improvement in the quality of life [42].

## 10. Reactive Treatment of Skin Toxicity

Despite vast publications on expert experience regarding the optimal treatment of EGFRI induced skin toxicity, they are mainly based on a few small studies and anecdotal reports. Therefore, evidence-based treatment recommendations are lacking.

Kanazawa et al. tested the effect of aspirin on the management of skin toxicity from gefitinib. In this study, gefitinib was given solely for the first 2 years in the first group. Then in the following 2 years, gefitinib was administered concomitantly with low dose aspirin (100 mg per day) in the second group. While there was no difference in therapeutic response in the two groups, the frequency of rash was significantly higher in the nonaspirin group [43].

Wong et al. evaluated the effect of Regenecare gel composing 2% lidocaine, aloe vera, marine collagen, and sodium alginate on skin toxicity induced by various types of EGFRIs. Regenecare gel was applied to the right side of the face for 1 week and later applied to the entire face. There was a significant improvement in itchiness. However, the authors did not provide any information about its impact on skin toxicity [44].

Vitamin K1 cream was administered as the management of cutaneous side effects from EGFRI in several uncontrolled studies. The first was a study by Ocvirk and Rebersek. Vitamin K1 cream was given twice daily to patients treated with cetuximab in combination with other chemotherapies after the first document of skin toxicity. All patients had improvement of cutaneous toxicity with downstaging in rash of at least one grade in 18 days [45]. Pinto et al. conducted another study where vitamin K1 cream was applied at the first onset of Grade ≥2 rash on patients receiving cetuximab or panitumumab. Oral tetracycline was also given in conjunction to vitamin K1 in 39.4% of the patients. 36.4% of the patients showed decrease in skin rash from Grade 0 to 1, 39.4% showed unchanged grading, and the rest had increase in grading to Grade 3. Good rash associated symptoms were obtained in the majority of patients [46].

The effectiveness of topical nadifloxacin cream and prednicarbate cream on acneform eruptions from cetuximab was evaluated by Katzer et al. This was an uncontrolled, open labeled study where nadifloxacin and prednicarbate cream were applied once daily on the skin lesions. The authors reported significant improvement in papules, pustules, and erythema at all-time points of evaluation [47].

Anecdotal reports of the success of retinoids on cutaneous toxicity from EGFRI have been published. Acetretin has been reported to improve erlotinib induced papulopustules [48]. Oral isotretinoin was a successful treatment for acneform skin lesions associated with cetuximab [49]. Application of adapalene reduced severe acneform eruptions from cetuximab [50].

Taking published trials into account, prophylactic systemic tetracycline and tetracycline class antibiotics have proven to be most effective. Avoidance of prolong sun exposure and application of sunscreen along with moisturizing cream and gentle cleansers, although lacking evidence, should still be considered as general patient recommendations.

## 11. Conclusions

In the era where administration of targeted monotherapeutic agents was increasingly popular, EGFRI have shown to have enormous cutaneous toxicity. It is important for physicians and dermatologist to recognize the wide variety of skin adverse events as well as give best possible treatment. Prophylactic measures give promising results, particularly oral tetracycline and tetracycline class antibiotics. Standard studies-based therapies are lacking. Optimizing management will continue to gain importance because it will allow these patients to remain on this life-saving targeted chemotherapy.

## Figures and Tables

**Table 1:** Summery of oral antibiotic in the prevention of EGFRI-induced skin toxicity.

Author Year	EGFRI agent	Patients (*n*)	Antibiotic	Objective	Results	Quality of life
Scope et al. 2007 [35]	Cetuximab	48	Minocycline	To decrease or prevent skin toxicity	Lower facial lesion count with minocycline (*P* value 0.05)	Lower itch severity
Jatoi et al. 2008 [36]	Multiple	61	Tetracycline	To prevent or decrease grade ≥2 rash	No difference in rash incidence (70% versus 76% *P* value 0.61) Significant lower grade ≥2 rash (17% versus 55%, *P* value 0.04)	Less burning and irritation with tetracycline
Laouture et al. 2010 [37]	Panitumumab	95	Doxycycline (plus skin moisturizer, sunscreen, and topical steroid) as prophylactic regimen	To decrease grade ≥2 toxicity	Lower incidence of grade ≥2 toxicity in prophylactic regimen (29 versus 62%, OR, 0.3; 95% CL, 0.1 to 0.6)	More improvement of DLQI in prophylactic group
Deplaque et al. 2010 [38].	Erlotinib	147	Doxycycline	To prevent or decrease severity of folliculitis	No difference in folliculitis incidence (68% versus 82%, *P* value = 0.055). Significant decrease in severity *P* < 0.001 Lower incidence of grade ≥2 folliculitis in doxycycline arm (39% versus 82%)	NA

All are RCTs.

DLQI: Dermatologic Life Quality Index. NA: not assessed.

**Table 2:** Summery of topical treatment in the prevention of EGFRI-induced skin toxicity.

Author Year	Type of study	EGFRI agent	Patients (*n*)	Topical agent	Objective	Results	Quality of life
Scope et al. 2007 [35]	RCT	Cetuximab	48	Tazarotene applied to half of the face	To decrease or prevent skin toxicity	No difference in the two groups	32.6% discontinued tazarotene due to significant irritation
Ocvirk et al. 2008 [41]	Uncontrolled trial	Cetuximab	43	Vitamin K1 cream	To decrease or prevent skin toxicity	65% developed skin toxicity, limited to merely grade 1 and 2	NA
Scope et al. 2009 [40]	RCT	Cetuximab	24	Pimecrolimus applied to half of the face	To decrease or prevent skin toxicity	Decrease lesion count in pimecrolimus treated side *P* value < 0.001 in week 2 *P* value = 0.02 in week 5	NA
Jatoi et al. 2010 [42]	RCT	Multiple	110	Sunscreen with SPF of 60	To decrease or prevent skin toxicity	No difference in rash incidence (72% versus 80% *P* = 1.00) or severity	No difference in quality of life

NA: not assessed.
